# Role of Immunogenetics in the Outcome of HCMV Infection: Implications for Ageing

**DOI:** 10.3390/ijms20030685

**Published:** 2019-02-05

**Authors:** Anna Aiello, Giulia Accardi, Giuseppina Candore, Calogero Caruso, Claudia Colomba, Danilo Di Bona, Giovanni Duro, Caterina Maria Gambino, Mattia Emanuela Ligotti, Janardan P. Pandey

**Affiliations:** 1 Sezione di Patologia Generale, Dipartimento di Biomedicina, Neuroscienze e Diagnostica Avanzata, Università di Palermo, Corso Tukory 211, 90134 Palermo, Italy; anna.aiello@unipa.it (A.A.); giulia.accardi@unipa.it (G.A.); giuseppina.candore@unipa.it (G.C.); caterinamaria.gambino@unipa.it (C.M.G.); mattiaemanuela.ligotti@unipa.it (M.E.L.); 2 Dipartimento di Scienze per la Promozione della Salute e Materno-Infantile, di Medicina Interna e Specialistica di Eccellenza “G. D’Alessandro”, Università di Palermo, Via del Vespro 129, 90127 Palermo, Italy; claudia.colomba@unipa.it; 3 Dipartimento dell’Emergenza e dei Trapianti d’Organo, Università di Bari Aldo Moro, Piazza G. Cesare 11, 70124 Bari, Italy; danilo.dibona@uniba.it; 4 Istituto di Biomedicina e Immunologia Molecolare, Consiglio Nazionale delle Ricerche, Via Ugo La Malfa 153, 90146 Palermo, Italy; giovanni.duro@ibim.cnr.it; 5 Department of Microbiology and Immunology, Medical University of South Carolina, 171 Ashley Ave, Charleston, SC 29425, USA; pandeyj@musc.edu

**Keywords:** antibodies, elderly, GM, immunosenescence, HCMV, HLA, NK, KIR

## Abstract

The outcome of host-virus interactions is determined by a number of factors, some related to the virus, others to the host, such as environmental factors and genetic factors. Therefore, different individuals vary in their relative susceptibility to infections. Human cytomegalovirus (HCMV) is an important pathogen from a clinical point of view, as it causes significant morbidity and mortality in immunosuppressed or immunosenescent individuals, such as the transplanted patients and the elderly, respectively. It is, therefore, important to understand the mechanisms of virus infection control. In this review, we discuss recent advances in the immunobiology of HCMV-host interactions, with particular emphasis on the immunogenetic aspects (human leukocyte antigens, HLA; killer cell immunoglobulin-like receptors, KIRs; immunoglobulin genetic markers, GM allotypes) to elucidate the mechanisms underlying the complex host-virus interaction that determine various outcomes of HCMV infection. The results, which show the role of humoral and cellular immunity in the control of infection by HCMV, would be valuable in directing efforts to reduce HCMV spurred health complications in the transplanted patients and in the elderly, including immunosenescence. In addition, concerning GM allotypes, it is intriguing that, in a Southern Italian population, alleles associated with the risk of developing HCMV symptomatic infection are negatively associated with longevity.

## 1. Introduction

Both environmental and genetic factors, as well as factors related to viruses, play a key role in determining the outcome of viral infections. Therefore, different individuals vary in their relative susceptibility to infections [1]. Regarding the genetic component of the response to viruses, the genes involved are polymorphic most likely because of Darwinian selection. Indeed, as suggested for the first time by J.B.S. Haldane, the main infectious diseases were the most important selective forces in shaping our evolutionary history [2].

It is well known that human cytomegalovirus (HCMV) is an important pathogen from a clinical point of view, because it causes significant morbidity and mortality in immunosuppressed individuals such as the transplanted patients and the elderly, in turn exacerbating their immunosenescence [3,4]. It is, therefore, important to understand the underlying mechanisms of virus infection control.

HCMV is a member of the herpes virus family (type 5) that is ubiquitous in human populations. HCMV, the largest human virus, has 235 Kb, double-stranded linear DNA genome. HCMV is transmitted from human to human. There is no animal reservoir. The transmission occurs via saliva, urine, breastfeeding, placenta, sexual intercourse, blood and organ transplants. In transplanted patients, immunosuppressive drugs can increase the risk of infection and complications [4,5,6,7].

Clinically, primary HCMV infection is asymptomatic in the 90% of the infected and immunocompetent population. Symptomatic primary HCMV infection of the adolescent or adult usually results in a mononucleosis-like syndrome with malaise, fever, sweating, and abnormal liver function [8]. In any case, a latent infection that lasts a lifetime is established. In a review of literature, seroprevalence was found to range from 45% to 100%, being the highest in South America, Africa and Asia and the lowest in Western Europe and United States. Worldwide, seroprevalence among whites tended to be 20–30 percentage points lower than that of Caucasians. Persons of higher socioeconomic status were more likely to be HCMV seronegative. In addition, seroprevalence increases with age, reaching more than 85% by 80 years. Although in most studies the differences were small, men generally had lower seroprevalences than women [3,7,9,10].

Most people carry the virus in a latent form and are at risk of reactivation. HCMV infection establishes a long-lasting immunity (but HCMV is able to manipulate the immune system) that limits the replication of virus after reactivation from latency. The reactivation mostly occurs both in immunosuppressed patients (as those transplanted) and in immunocompromised patients (septic patients or elderly) [11,12]. All these conditions are characterized by an inflammatory state, and it has been suggested that inflammation can be responsible for reactivation [13].

Pattern recognition receptors such as Toll-like receptors recognize components of the virion, thereby triggering the innate immune response to the virus with the production and/or release of pro-inflammatory cytokines that in turn recruit and activate cells of innate immunity, including Natural Killer (NK) cells that destroy target cells infected by HCMV. There is also triggering of acquired immunity that involves both T and B lymphocytes. It results in a large cytotoxic CD8 T cell response that plays a key role in the control of primary HCMV infection and reactivation from latency. In addition, T helper CD4 lymphocytes, through the production of interferon-γ and their helper function for CD8 and B lymphocytes, play a role in the control of HCMV infection [14]. Due to coevolution within their hosts for millions of years, herpes viruses, including HCMV, are able to encode several proteins and microRNAs responsible for evading the host immune response, both during lytic infection and during latency. It allows HCMV to replicate and disseminate in the face of a competent immune system and to establish latency with periodical viral reactivation and virus shedding through the life of the host. HCMV products are, indeed, able to inactivate complement cascade, to mitigate the effects of interferon and to prevent apoptosis of infected cells. Other virus products are proteins homologue for cytokines, chemokines and their receptors as well as for fragment crystallisable γ receptor (FcγR). Lastly, virus products interfere with T and NK cells functions. [15,16].

HCMV is not only a master in immune evasion but also is responsible for the manipulation of immune system with ageing. In the latent state, the intermittent production of viral antigens prevents contraction of virus-specific T cells. Thus, a large population of HCMV-specific CD8+, and to a lesser extent, CD4+ T cells, is generated. That is responsible for the phenomenon of memory cell inflation leading to the emergence of vast populations of resting effector CD8+ and CD4+ cells. In the elderly, many alterations of innate and acquired immunity have been described and viewed as deleterious, hence the term immunosenescence [17]. Age, followed by sex and the HCMV status, has the greatest impact on the immune system. HCMV is considered to contribute to shape the immune profile and function during normal human ageing. Accordingly, modulation of the immune response by HCMV may occur, resulting in less effective control of HCMV replication following virus reactivation [3,15,18]. However, inflationary CD8+ cells, after proper activation stimuli, can divide, secrete cytokines, and execute cytolysis, i.e., they are not exhausted. The production of inflammatory cytokines can contribute to the pro-inflammatory status of ageing, called inflamm-ageing [17]. Moreover, there is some evidence of a slight loss of control of HCMV replication in the elderly compared with younger people, as the HCMV load within blood increases markedly in healthy people over the age of 70 years [19]. There is some evidence of an association of HCMV seropositivity with increased mortality from cardiovascular disease, as a result of damage caused by the large expansion of cytotoxic CD4+CD28-negative T cell populations commonly seen in HCMV-infected individuals [20,21]. Immune changes associated with HCMV may have significant impact during co-infection and vaccination as well as on fitness [22,23]. Herpes virus latent infection should contribute to immune dysfunction in the elderly but may be beneficial during childhood-adult years [15,18].

In our review, we discuss recent advances in the immunogenetic control of HCMV-host interactions. We will focus on the role played by human leukocyte antigens (HLA) responsible for both humoral and cellular control, killer cell immunoglobulin-like receptors (KIRs), responsible for cellular control, after interaction with class I HLA, and γ marker (GM) immunoglobulin allotypes responsible for humoral control. Almost all of the papers concern the immunogenetic control of HCMV reactivation in transplanted patients. Data are also discussed considering the role of HCMV in ageing and immunosenescence.

## 2. HLA and HCMV

The HLA system encodes cell-surface proteins responsible for the regulation of the immune system. HLA genes are highly polymorphic, i.e., they have many different alleles, involved in fine-tuning of the acquired immune responses. HLA classes have different functions [24,25,26]. (I) Class I (HLA-A, -B and -C) presents peptides from inside the cell to the surface of the cell, including viral fragments if the cell is infected by a virus. It follows that the cell can be lysed by CD8+. (II) Class II (HLA-DRA, HLA-DRB1, HLA-DRB3–5, HLA-DQA, HLA-DQB, HLA-DPA and HLA-DPB) presents peptides from outside the cell to CD4+ cells, which in turn stimulate B lymphocytes to produce antibodies to that specific antigen. (III) Class III encodes components of the complement system and pro-inflammatory cytokines. The high polymorphism of class I and II molecules affects the peptide-binding groove, since it varies the amino acids sequences that can be housed within the peptide-binding pockets. Different HLA alleles possess different peptide-binding repertoires and it is difficult for disease-related proteins to escape detection. Therefore, it is not surprising that several infectious and autoimmune diseases are associated with different HLA antigens, responsible for different humoral or cellular immune responses [24,25,26]. However, in the last few years, it is becoming clear that Class I antigens can play a role as ligand for KIR. Therefore, several observed association of HLA class I antigen with infectious and autoimmune diseases can be explained by their role as ligands for KIR [26].

As mentioned previously, HCMV interferes with immune responses at different levels. Several proteins (or microRNA) produced by the virus can interfere with the functions of Class I and Class II molecules, i.e., antigen processing and presentation, e.g., they can induce their down regulation or their degradation [15,16].

Concerning the role of HLA in the control of HCMV infection, most studies regard HCMV infection/reactivation in patients after kidney transplantation. However, as reported by Futohi et al. [6] the results have been discordant. Therefore, no conclusion can be drawn from these studies, although in three papers out of eight an association with HLA-DR7 was reported [27,28,29].

As discussed by Caruso et al. [24], several methodological problems can explain the discrepancies observed in studies concerning association of HLA with diseases as follows. (I) Insufficient sample sizes to detect differences in HLA antigen frequencies. (II) Different inclusion with inappropriate mixing of data (cohort effect) and inappropriate control matching, i.e., lack of selection from the same target population. (III) The different genetic background of studied population. (IV) The lack of Bonferroni’s correction for multiple comparisons.

However, in the previously quoted study [6], the presence of HLA-B8 allele had a protective effect for developing HCMV infection after kidney transplantation. It is intriguing that in a study performed on HCMV infection in Ireland, HLA-A1, HLA-B8 alleles were associated with HCMV seronegativity [5]. These alleles are carried by the 8.1 ancestral haplotype, i.e., an evolutionarily highly conserved haplotype, known to influence cytokines production with decreased type 1 responses in contrast to type 2 ones. It follows that humoral responses are enhanced in people carrying this haplotype [30].

These results are of some importance for the relationship between HCMV and ageing. In fact, this haplotype has been associated with male longevity [24,25], hence this datum strengths the suggestion of the detrimental role for HCMV in achieving successful ageing.

## 3. KIR and HCMV

**Figure 1:**
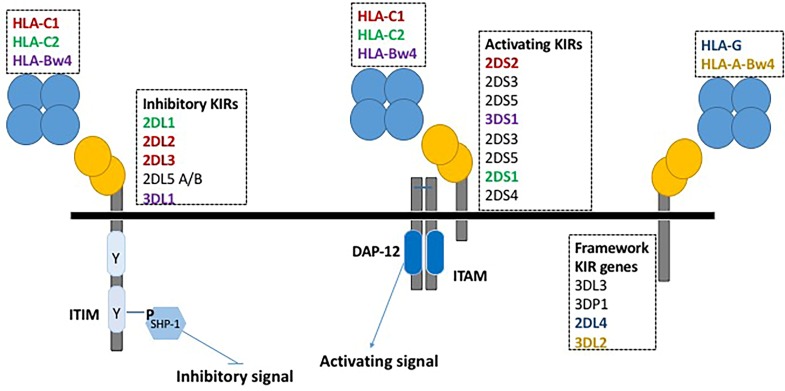
The figure shows the killer cell immunoglobulin-like receptor (KIR)/ human leukocyte antigens (HLA) interaction with inhibitory or activating effects. The activating receptors (↓) have short cytoplasmic tails lacking cytoplasmic immunoreceptor tyrosine-based inhibition motif (ITIM). The tails interact with the adaptor molecule, the death associated protein (DAP) 12, and contain immunoreceptor tyrosine-based activation motif (ITAM) linked to protein tyrosine kinase activation pathways. On the contrary, the inhibitory KIRs (⟘) are characterized by the presence of ITIM, which recruits the SH protein-1/2 tyrosine phosphatases. In the figure, the receptors, inhibitory or activating, and the HLA ligands, that interact are marked with the same colour. The ligands of the receptors marked in black are unknown. The interaction between KIR3DL2 and HLA-A-Bw4 is inhibitory. The four blue spheres depict the four domains of the HLA complex. The two yellow spheres depict the two immunoglobulin-like extracellular domains of KIR. However, the figure does not show KIRs that have three immunoglobulin-like extracellular domains.

KIRs, expressed on the membrane of NK cells and a minority of T lymphocytes, regulate the killing function of these cells by interacting with specific amino acid motifs, public epitopes, carried by some HLA class I molecules, and expressed on their targets. They are characterized by two or three extracellular domains and a short (S) or long (L) intracytoplasmic tail that respectively transduces either activating or inhibitory signals (see Figure 1). The KIR gene complex is characterized by multiple gene-content haplotypes, i.e., it is polygenic. KIR genes are organized in two basic haplotypes defined on the basis of gene content, and are termed A and B. Group A haplotypes are characterized by the absence of the following genes KIR2DL5, KIR2DS1, KIR2DS2, KIR2DS3, KIR2DS5 and KIR3DS1, whereas group B haplotypes are characterized by the presence of one or more of the previous genes. Thus, the B haplotypes have more genes encoding activating KIR than A haplotypes. Moreover, KIR genes exhibit allelic variability, i.e., they are polymorphic. Furthermore, KIR expression on NK cells is stochastic, resulting in a significant variation of the NK cell repertoire among individuals and among populations. KIRs are able to detect cells infected by viruses or transformed, by binding the different class I allelic variants. Most inhibitory KIRs specifically recognize sets of HLA class I alleles; yet, the ligands for some of them and for most activating KIRs are unknown. Specific combinations of KIRs with their cognate HLA ligands have been associated with infectious diseases, autoimmune disorders, pregnancy outcomes and cancer. In addition, they are crucial in determining clinical outcomes of both hematopoietic stem cell and solid organs transplantation [31,32,33,34].

There is compelling evidence that NK cells play a crucial role in host defence against HCMV infection. Thus, it is not unexpected the interference by proteins or microRNA coded by HCMV with NK function. As an example, several proteins affect the expression of ligands of the NKG2D receptor, a transmembrane protein encoded by KLRK1 activating gene (not linked to KIR genes) whose ligands are induced-self proteins completely absent or present at low levels on the surface of normal cells, but overexpressed in infected or transformed cells [35]. Clearly, the degradation or the down regulation of HLA Class I antigens, previously reported, interferes with KIR [15,16]. However, as shown in Figure 1, these antigens can bind both activating and inhibitory KIRs.

The entire population of NK cells from a child with a novel immunodeficiency syndrome and recurrent HCMV infection expressed the KIR2DL1, with inhibitory function. Since the patient also possessed the KIR2DL1 ligand group, HLA-C2, it was suggested the possibility that the strong inhibitory KIR2DL1/HLA-C2 combination impaired NK cell activity and prevented the cells from mounting a protective response against HCMV [36].

Several reports have documented a role for activating KIRs in the control of HCMV infection after hematopoietic stem cell or kidney transplantation. Activating KIR genes carried by kidney transplant-recipients have been shown to influence the rate of viral infection during the first year after transplantation. In fact, patients carrying more than one activating KIR gene (KIR BA or BB genotypes) showed a rate of infection and reactivation significantly lesser (20%) than that observed in patients with a KIR AA genotype (36%) (with KIR2DS4 as only activating KIR). Moreover, the number of activating KIR genes correlated with the degree of protection. This study supports a role for activating KIR in the control of HCMV infection after kidney transplantation [37]. The KIR/HLA genotypes and the rate of HCMV infection was analysed in 196 kidney transplant recipients. In kidney recipients, it was shown that, after transplantation, both the presence of activating KIR genes and the absence of the HLA-C ligands for inhibitory KIR were associated with a lower rate of HCMV [38]. After hematopoietic stem cell transplantation, whether the donor had either ≥5 activating KIR genes or KIR2DS2 and KIR2DS4 genes, there was a low risk of HCMV reactivation in the recipient [39]. Finally, in stem cell transplantations involving siblings where both donor and recipient were HCMV seropositive and the donor possessed a copy of the KIR haplotype B, the reactivation rate was 28%, whereas the HCMV reactivation rate was 65% if donor was homozygous for the KIR A haplotype, i.e., with only one activating KIR. A significant decreased risk of HCMV reactivation was also confirmed by multivariate analysis if the donor had more than one activating KIR gene [40]. All these results obtained in transplanted patients demonstrate the importance of activating KIRs in the immune surveillance against HCMV.

Interestingly, similar data were obtained in immunocompetent subjects, studying a cohort of 120 subjects with HCMV infection [41]. Indeed, evidence has been provided for a detrimental effect of the AA haplotype or the HLA-Bw4^T^ group on the outcome of primary HCMV disease. The frequency of the homozygous A haplotype was higher in symptomatic patients than in controls. By logistic regression, the risk of developing symptomatic disease was associated with the homozygous A haplotype and the HLA-Bw4^T^ groups. These results show the key role played by KIR/HLA polymorphisms in the immunocompetent host, suggesting that HLA-Bw4^T^ group exerts its function by binding the inhibitory KIR3DL1 gene. With the aim to gain insight in the association of this combination and HCMV, our group analysed the distribution of HLA-B alleles, in symptomatic patients carrying HLA-Bw4^T^ group. We observed (Table 1) that, in symptomatic patients carrying HLA-Bw4T group, HLA-B*44 allele was over-represented, 55.6%, whereas its frequency in Sicily is 8.9% [42]. This result is intriguing since this allele is in linkage disequilibrium with HLA-DR7 [43], reported to be associated with infection/reactivation in patients after kidney transplantation [27,29].

Finally, the correlation of KIR gene distribution and the anti-HCMV antibody titer has been studied in the elderly. Analysis of the distribution of KIR genes showed a non-significant decreased frequency of inhibitory KIR2DS5 gene in the group with higher (>20 IU/mL) HCMV-specific IgG antibody levels [44,45]. Therefore, a study in a larger group of elderly is warranted.

## 4. GM Allotypes and HCMV

The term allotype refers to any genetic variant of a protein. In immunology, GM allotypes indicate allelic hereditary variants, encoded by autosomal codominant alleles that follow Mendelian laws of heredity, expressed on immunoglobulin constant region of γ1, γ2 and γ3 chains. GM allotypes are encoded by three very closely linked, highly homologous, immunoglobulin heavy gamma (IGHG) genes, on chromosome 14q32. Linkage disequilibrium in the GM system within an ethnic group is almost absolute and the determinants are transmitted as a group, i.e., haplotype. Each major ethnic group has a distinct array of several GM haplotypes [46,47].

These observations point towards a role for differential selection in the maintenance of GM polymorphism. Many lines of evidence point towards infectious diseases as the principal selective forces of natural selection [2]. GM allotypes have been shown to be associated with immunity to many infectious pathogens. They also influence the chance for survival from epidemics, such as typhoid and yellow fever [48]. Different mechanisms have been proposed to explain these associations [49].

There are inter individual differences in the level of anti-HCMV IgG antibodies, suggesting the existence of host immune response genes for this trait [50]. However, a genome-wide association study (GWAS) found no major genes for anti-HCMV antibody responsiveness [51]. As pointed out elsewhere [52,53], current GWAS do not evaluate GM genes because they are not included in the commonly used genotyping arrays. The extensive homology of IgG gene segments expressing various GM allotypes may have contributed to their exclusion from these arrays. Therefore, it is necessary to employ a candidate gene approach for evaluating the role of GM genes in the immunobiology of HCMV infection.

Using a candidate gene approach, the contribution of GM allotypes to the magnitude of antibody responses to HCMV glycoprotein B (gB), which is required for viral infectivity and is a major component of the viral envelope, was investigated. Results showed that two allotypes at the γ1 locus, GM3 and GM17, additively contributed to the level of IgG antibodies to gB. The homozygosity for the GM 17 allotype was associated with high, while the homozygosity for its allelic counterpart (GM 3) with low, anti-HCMV gB antibody levels, respectively. The heterozygotes exhibited intermediate levels of antibodies. GM 5 and GM 21 allotypes, which are in linkage disequilibrium with GM 3 and GM 17 allotypes, respectively, followed a similar pattern of anti-HCMV gB antibody responses [50].

Several mechanisms could account for the GM gene involvement in humoral immunity to HCMV: structural contribution to the idiotypes involved in HCMV immunity, contribution to the conformational modifications of antibody binding sites that could influence its affinity, and linkage disequilibrium of constant-region GM allotypes with the variable-region genes. Murine studies have clearly shown that differences in the amino acid sequences in the constant region affect the secondary structure of the antigen-binding site in the variable region. Amino acid substitutions that characterize GM allotypes cause structural changes in the constant region, which could impose structural constraints (conformation) on the variable region, resulting in variation in antibody affinity to HCMV. Thus, isotype restriction of antibodies to HCMV, which are predominantly IgG1 and IgG3, may reflect structural constraints imposed by the constant-region allotypes on the variable-region binding [46,49,54].

As previously stated, the HCMV has developed a large repertoire of highly sophisticated strategies, directed against almost every component of the human immune system, for evading host immunosurveillance [15,16]. One of these strategies involves the generation of three proteins, encoded by genes TRL11/IRL11, UL119-UL118 and RL13 that have functional properties of the FcγR [16,55]. The viral FcγRs essentially neutralize the constant region of anti-HCMV IgG antibodies that are involved in effector functions, such as antibody-dependent cellular cytotoxicity, antibody-dependent cellular phagocytosis and complement-dependent cellular cytotoxicity. This helps the virus to evade host immunosurveillance. GM allotypes have been shown to modulate this viral immunoevasion strategy. IgG1 proteins expressing the haplotype carrying GM3 allotype had significantly higher affinity for the TRL11/IRL11-encoded decoy FcγR than IgG1 expressing the haplotype carrying GM17 allotype. In contrast, the UL119-UL118-encoded FcγR has higher affinity for the IgG1 proteins expressing the GM17 haplotype than those expressing the GM3 haplotype. The affinity of the RL13-encoded FcγR to the allotypically disparate IgG1 proteins follows a similar pattern [56,57,58].

An interaction between HLA/KIR genes and GM allotypes in the control of HCMV infection was shown in Sicilian population. Particularly, it was shown the association of GM17/17 with the risk of developing symptomatic infection with a gene–dose effect, i.e., the association was observed only in homozygous people. The risk of symptomatic infection was also shown in people carrying GM23, without evidence of a gene–dose effect. In addition to these GM allotypes, HLA-C2 and HLA-Bw4^T^ groups remained independently associated with the risk of HCMV symptomatic infection in a multiple logistic regression analysis. Therefore, both a possible epistatic interaction of GM allotypes with HLA gene variants, and an independent effect of these GM allotypes can be hypothesized [59]. These results are not in contrast with the above reported association of GM17 allotype with an increased antibody response to HCMV [50], because this increased response should depend on a less efficient control of HCMV that determines a greater antigenic stimulation.

Regarding the relationship between HCMV and ageing, it is noteworthy that, in another Southern Italy population, GM3 allotype, the alternative allele of GM17, was significantly overrepresented in long-living individuals [60], strengthening the suggestion of the detrimental role for HCMV in achieving successful ageing.

To confirm and extend these observations, large scale studies involving GM allotypes and anti-HCMV antibodies should be conducted in long-living individuals and young controls.

## 5. Summary

Considering the profound effects of HCMV infection on health and the quality of life of immunosenescent individuals—either transplanted patients or old subjects—the identification of the mechanisms responsible for the immunogenetic control of HCMV infection should have important clinical implications. Therefore, the data discussed on the role of humoral and cellular immune responses in the control of HCMV infection are important in driving efforts to reduce the HCMV-associated health complications in old subjects and in transplanted patients. Further efforts in determining the crucial role of HLA, KIR and GM genes in HCMV control will greatly enhance our understanding of the immunogenetic aspects of HCMV infections, and to potentially apply this knowledge clinically, i.e., in the development of new vaccines and in the identification of new therapeutic targets.

## Figures and Tables

**Table 1:** Distribution of HLA-B alleles in Human cytomegalovirus (HCMV) symptomatic patients positive for the different HLA-Bw4 groups. (The number of alleles is twice the number of subjects, as each person has two alleles—one maternal and one paternal).

HLA-Bw4 Groups	HCMV Patients (*N* = 31)	
	N	%
**HLA-Bw4^T^**		
B*44	11	33.30
B*13	4	12.12
B*35	3	9.09
B*14	1	3.03
B*40	1	3.03
B*27	2	6.06
B*73	1	3.03
B*50	1	3.03
B*07	0	0
B*18	0	0
**HLA-Bw4^I^**		
B*27	1	3.03
B*14	1	3.03
B*39	1	3.03
B*57	3	9.09
B*35	5	15.15
B*38	2	6.06
B*40	1	3.03
B*49	3	9.09
B*50	2	6.06
B*08	1	3.03
B*15	1	3.03
B*51	4	12.12
B*67	1	3.03
B*18	0	0
B*53	1	3.03
B*58	3	9.09
B*44	1	3.03
B*52	1	3.03
**HLA-Bw4^T^/HLA-Bw4^I^**		
B*44	4	12.12
B*13	0	0
B*49	1	3.03
B*53	1	3.03

Genomic DNA was extracted by a commercial kit (PureLink^®®^ Genomic DNA, ThermoFisher Scientific, Waltham, MA, USA) from frozen mononuclear cells obtained in the previous study [41] from peripheral whole blood samples. The HLA-B loci genotypes were determined using the commercially available HLA Class I B Locus DNA Typing Tray kit (One Lambda, ThermoFisher Scientific Brand, California, USA), according to the manufacturer instructions.

## Abbreviations

FcγR	fragment crystallisable γ receptor
gB	glycoprotein B
GWAS	genome-wide association study
GM	genetic marker
HCMV	human cytomegalovirus
HLA	human leukocyte antigens
IGHG	immunoglobulin heavy gamma
KIRs	killer cell immunoglobulin-like receptors
NK	natural killer
